# Study of the right ear advantage on gap detection tests

**DOI:** 10.1016/S1808-8694(15)31094-6

**Published:** 2015-10-19

**Authors:** Alessandra Giannella Samelli, Eliane Schochat

**Affiliations:** 1 Doctor in Science, FMUSP, faculty member of the speech therapy course, Guarulhos University; 2 'Livre-docente' habilitation, faculty member of the speech therapy course, FMUSP

**Keywords:** auditory cortex, laterality, auditory perception

## Abstract

Temporal resolution hearing skills are based on the minimum time necessary to segregate or solve acoustic events. This skill is fundamental for speech comprehension and can be assessed by gap detection tests. Some studies point to a right ear advantage over the left ear in temporal resolution tasks, since there is a preferential role of the left hemisphere in analyzing the temporal aspects of the acoustic stimulus.

**Aim:**

determine if there are response differences (gap detection thresholds and percentage of correct answers) between right and left ears in a gap detection test. Study: experimental.

**Materials and Methods:**

the gap detection test was applied to 100 adult individuals, after carrying out other audiologic tests in order to rule out possible hearing and/or auditory processing disorders.

**Results:**

We observed gap detection thresholds and average correct answers percentages, which were similar for both ears, regardless of which ear started the test.

**Conclusion:**

There was no ear advantage in the gap detection task.

## INTRODUCTION

The gap detection test is a relatively simple psychoacoustic test for measuring temporal resolution.^1^, ^2^, ^3^, ^4^, ^5^, ^6^, ^7^ The auditory ability for temporal resolution is the minimum time required for separating or resolving acoustic events.^8^^,^^9^

In trying to locate the physiological mechanism of temporal resolution, some authors have suggested that auditory nerve fibers participate significantly in the process.^5^^,^^10^^,^^11^ Other studies, however, have shown that processing is more central.^12^, ^13^, ^14^, ^15^, ^16^, ^17^, ^18^, ^19^, ^20^, ^21^, ^22^, ^23^, ^24^, ^25^, ^26^

Temporal resolution depends on the separation of different auditory stimuli; the role of the initial part of the stimulus and the coding precision of this response are crucial.^19^^,^^24^

Auditory cortex neurons are particularly sensitive to these initial transitory stimuli, including the beginning of periodic events, the incident modulation of other periodic signals o the acoustic events that occur naturally in vocalization.^12^

Primary auditory cortex neurons respond briefly and transitorily to the beginning of sounds, regardless of signal duration. These neurons are sensitive to sound frequency and the duration of its onset (attack), which contributes significantly to the signal onset short-term spectrum. Response brevity at the onset of sound is marked by the post-initial inhibitory response and by neural adaptation.^15^

The precision of the first neural firing that responds to the onset of sound is proportional to the neural response latency. Phillips and Hall^13^ found latency as short as 0.45 −1.5 ms for first neural firing in the auditory cortex of cats. These values are very close to those observed in the cochlear nerve and the cochlear nucleus, suggesting that temporal fidelity for transitory responses is preserved in afferent pathways until the primary auditory cortex.^17^^,^^22^

This degree of precision in response time supports temporal resolution at the limits of behavioral performance, a task for which the auditory cortex is important. Furthermore, such degree of precision is able to represent the duration of the phonetically important components of speech signals.^13^^,^^16^

The human auditory cortex is located in the temporal lobe. It is organized in a koniocortical cytoarchitecture central core (small cells in all layers in a highly granulated and myelinized area) surrounded by a less granulated cortical auditory belt. This core area is the primary auditory cortex, located in the transverse gyrus (Heschl's gyrus) on the upper surface of the temporal lobe.^27^

Heschl's gyrus is highly variable among individuals and between both hemispheres. It may contain one to three gyruses per hemisphere and the number of gyruses is not necessarily equal in both hemispheres.^28^

The primary auditory cortex is located approximately in half of the first gyrus or in half of the first gyrus and in part of the second gyrus; it covers about the central two thirds of Heschl's gyrus.^25^^,^^28^, ^29^, ^30^

Many studies have demonstrated the existing asymmetry between the right and left Heschl's gyruses. The left gyrus is larger than the right gyrus; thus, the left primary auditory cortex is also larger than the right primary auditory cortex. The increased left size volume results from more gray and white matter present in this side.^28^^,^^31^^,^^32^

The larger neural substrate (more neurons and more intra- and interhemispheric interconnections) in these left hemisphere anatomic structures provides the basis for better language development compared to the corresponding (smaller) areas in the right side.^32^

Left hemisphere speech specialization may be related to an identification of specific acoustic parameters for sound and speech discrimination. The ability to code and analyze temporal aspects within acoustic information may be related with the left hemisphere contribution to language functions.^28^

Numerous findings have demonstrated the preferential role of the left hemisphere in analyzing temporal aspects of acoustic stimuli; it is possible that the observed structural differences between both hemispheres define this differentiated ability.^28^^,^^33^

Zatorre and Belin^33^ investigated functional differences between the auditory cortex of the right and left hemispheres. Neuroimaging methods revealed increased activation of Heschl's gyrus in both hemispheres, although the left side responded more to temporal tasks; on the other hand, spectral changes caused more activation in the superior temporal gyrus bilaterally, but with an increased response in the right side. These differences were explained as being anatomical differences. More significant left hemisphere myelinization increases conduction speed, making this hemisphere more sensitive to rapid acoustic changes. At the same time, increased space between cortical columns and highly intrinsic connections in the left hemisphere allow integration along tonotopically-organized areas, impoverishing the spectral resolution. The opposite applies to the right hemisphere, in which structural patterns favor high frequency resolution at the cost of slower transmission.

Brown e Nicholls^34^ used a gap detection test to assess adult temporal resolution and perceptual asymmetry between ears. The acoustic stimulus was broadband noise (74 dB NPS) lasting 300ms. Four gap intervals were inserted: 2, 4, 6 and 8ms. The authors found faster and more accurate responses in the right ear (left hemisphere) compared to the left ear.

Sulakhe et al.^35^ obtained similar results. Two types of noise (white and narrow band noise) were used. Stimulus duration was 300ms and the gaps were 3, 4 or 5ms. The authors found hemispheric asymmetry with a right ear advantage for white noise and hemispheric symmetry for narrow band noise. The explanation was that these differences (symmetry versus asymmetry) could be attributed to the various stimulus parameters.

Other authors, however, have found no perceptual asymmetry between right and left ears, that is, they observed no right ear advantage over the left ear in auditory temporal processing and temporal resolution tasks.^36^, ^37^, ^38^

The purpose of this article was to observe whether there were any response differences (gap detection thresholds and percentage of correct answers) between right and left ears in a gap detection test.

## MATERIAL AND METHOD

The Research Ethics Committee for research project analysis (CAPPesq) approved this study (protocol number 113/02, 13/03/2002).

### Subjects

The analysis included 100 adult subjects aged from 18 to 31 years; 50 were male (mean age 24.72 years) and 50 were female (mean age 23.77 years). The youngest male was aged 18 years and the youngest female was aged 18.16 years. The maximum age in males was 31.5 years and in females it was 31.83 years (p=0.176).

The sample was not divided according to right-handedness, as the general prevalence of right-handed individuals is about 90%. Furthermore, 95% of right-handed and 70% of left-handed individuals have left hemispheric language specialization.^39^ If the sample contained individuals with right hemispheric specialization, they would not be sufficient to contaminate the results.

### Material and Procedures

All participants were informed about the voluntary nature of the study, its objectives, the exams that would be applied, the absence of health risks due to the study procedures, and the dissemination of results among the science community. If there was agreement about these requirements, patients were asked to sign a free informed consent form before participating in the study.

After this agreement, the following procedures were undertaken in the entire sample: clinical history taking, pure tone audiometry, immitance testing and the digital dichotic test (as basic screening of auditory processing^40^). All subjects had auditory thresholds below 20 dBNA at all of the tested frequencies (0.25 to 8 KHz),^41^ a type A tympanometric curve and ipsi- and contralateral acoustic reflexes, and correct answers equal to or over 95% in each ear in the digital dichotic test.^44^

The GIN test, developed by Musiek in 2003,^38^^,^^45^ was then applied. An Interacoustic AC 40 audiometer, coupled to a Sony CD player, in an acoustic booth, at a 50 dB NS intensity (according to mean auditory thresholds at 500, 1000 and 2000 Hz in each ear) was used for the test. A monaural presentation condition was used throughout.

The GIN test is composed of various 6-second white noise segments, each with 0 to 3 silence gaps each. Noise segments are separated from each other by a 5-second silent interval (interval between stimuli) and gap duration is 2, 3, 4, 5, 6, 8, 10, 12, 15 and 20ms. The occurrence of gap duration and location within noise segments is pseudo-randomized. There are ten practice items that precede the beginning of test items. Each gap of different duration appears six times in each list. The test is composed of four lists.^38^

Each ear is assessed separately twice (two lists for each ear). Two measurements are made for each list: gap detection threshold (smallest gap perceived by patient in at least 50% of presentations - three times - as each gap appears six times in each list), and the percentage of correct answer in each list (the total number of gaps detected).

Subjects were divided as follows to discard possible influences from the test ear: the GIN was done first in the right ear in 50% of subjects (25 male subjects and 25 female subjects), and the GIN was done first in the left ear in the other 50% of subjects (25 male subjects and 25 female subjects).

The ANOVA test was used for the statistical analysis. The significance level was 0.05.

## RESULTS

**Figure 1:**
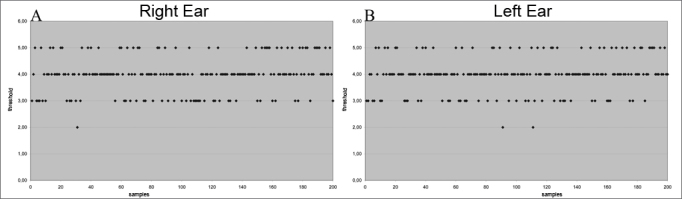
Gap detection thresholds in 400 samples (in ms) - (A) right ear (B) left ear.

Right and left ear results of the 100 subjects are presented regardless of the first ear that was tested. As each ear was assessed twice, 400 samples were collected, 200 from right ears and 200 from left ears (Figures 1 A and 1B).

Figures 1 A and 1B show that gap detection thresholds were similar in both ears. Furthermore, most of the thresholds were about 4ms for both ears.

These results are made more evident by the statistical analysis (ANOVA) (Table 1).

**Table 1:** Mean, standard deviation and the p-value for gap-detection thresholds in a comparison between right and left ears.

Ear	n	Mean (ms)	Standard deviation	p
RE	200	3,985	0,683	1,0000
LE	200	3,985	0,669	

**Figure 2:**
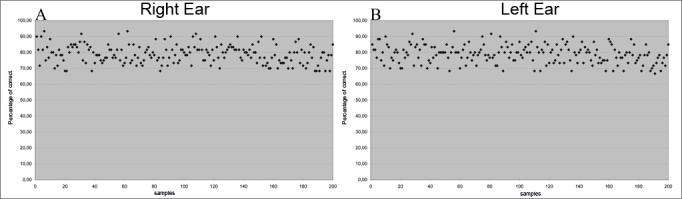
Percentage of correct answers in 400 samples (in %) - (A) right ear (B) left ear.

A similar pattern was observed in the percentage of correct answers, in which right and left ear results were similar (Figure 2 A and 2B; Table 2).

**Table 2:** Mean, standard deviation and the p-value for the percentage of right answers in a comparison between right and left ears.

Ear	n	Mean (%)	Standard deviation	p
RE	200	78.922	5.810	0.8650
LE	200	78.872	5.836	

**Figure 3:**
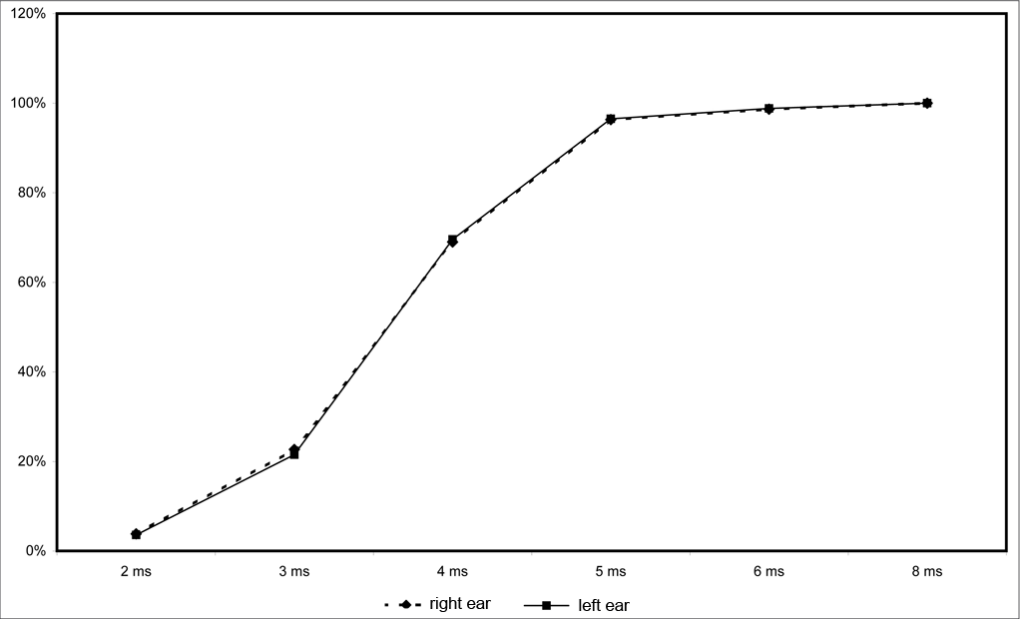
Percentage of correct answers per gap intervals in each ear (in %).

Figure 3 shows the correct answers in each ear for gap intervals of 2 to 8ms. The percentage of correct answers for these gap intervals are similar in both ears; there was no higher prevalence of correct answers in any one ear. The percentage of correct answers for a 2ms gap interval was close to 4%. This percentage was around 20% for 3ms. Correct answers improved considerably (around 70%) for a 4ms gap interval. The percentage of correct answers was higher than 90% for 5 and 6ms gap intervals, reaching 100% for 8ms or more gap intervals.

## DISCUSSION

These results reveal that no ear had any advantage relative to the other in all of the tests (gap thresholds, percentage of correct answers or percentage of correct answers per gap interval).

These results differ from those in various other published papers, which have shown an advantage for the right ear (left hemisphere) in temporal resolution tasks.^34^^,^^35^ Our results, however, agree with those of Efron et al.,^36^ Baker et al.,^37^ and Musiek et al.,^38^ in which no perceptual asymmetry was found between ears in gap detection procedures. Baran and Musiek^46^ have stated that monochotic tests are useful for detecting alteration in auditory pathways but not for locating these changes, as ipsi- and contralateral pathways participate in this process, which results in similar right and left ear performance.

Many authors have suggested that the left hemisphere has a preferential role in analyzing temporal aspects of acoustic stimuli.^28^^,^^33^

The currents study found no right or left ear advantage (or no hemispheric advantage) in gap detection tasks. There are, however, a few points that should be emphasized.

Brown and Nicholls^34^ and Sulakhe et al.^35^ have reported a right ear advantage over the left ear, while Efron et al.,^36^ Baker et al.,^37^ and Musiek et al.^38^ found no asymmetry between ears.

Brown and Nicholls^34^ and Sulakhe et al.^35^ used the reaction time to the presence of gaps for their analysis. Further more, the former study evaluated the rate of false results, while the latter assessed the percentage of correct answers. The reaction time was not tested in the current study and in Efron et al.'s^36^ and Baker et al.'s^37^ studies, which may in part explain the difference between results. If the left hemisphere has a larger neural substrate,^32^ a more rapid right ear temporal resolution might be expected, as found by the abovementioned authors.

The type of response in these studies was a “yes or no,” procedure, while Efron et al.^36^ and Baker et al.^37^ used the 2AFC (two-alternative forced-choice) procedure, as well as different analysis parameters, namely the psychometric function (percentage of correct answers per gap interval), similar to the current study.

Gap thresholds and the percentage of correct answers might possibly mask the right ear advantage, which may become even more evident in the reaction time analysis.

In summary, different parameters used in these studies, including this one, may explain these contradictory findings on the perceptual asymmetry of temporal resolution between ears. This does not mean, that there is no left hemisphere advantage for such tasks, but rather that certain procedures cannot assess this difference. It should also be borne in mind that ipsi- and contralateral pathways are activated in monochotic assessments, which precludes advantages for any ear.

Furthermore, other cortical areas - besides the primary auditory cortex - may participate in auditory processing of rapid stimuli; further conclusions are not possible without more detailed investigation about this theme.^47^^,^^48^

## CONCLUSION

There was no difference in gap-detection thresholds and the percentage of correct answers between right and left ears in the gap detection test (GIN - Gaps In Noise).
